# Epigenetic Regulation of Cell Cycle Control in Colorectal Cancer: Molecular Mechanisms and Therapeutic Perspectives

**DOI:** 10.34172/apb.47190

**Published:** 2026-05-23

**Authors:** Srinivasa B, Devika P Jeeragyal, Kiran Velukuri, Amarnath Reganti, Vaheedha S, Vinodhkumar Ramalingam, Rajasekar Sreerama, Neelima Grandhe, Praveen Hoogar

**Affiliations:** ^1^Department of Pharmacology, Saveetha Medical College & Hospital, Saveetha Institute of Medical and Technical Sciences (SIMATS), Saveetha University, Chennai Tamil Nadu, India; ^2^Department of Community Medicine, Apollo Institute of Medical Sciences And Research Chittoor, India; ^3^Department of Physiotherapy, Apollo College of Physiotherapy, the Apollo Knowledge Centre, Murukambattu, Chittoor 517127, India; ^4^Division of Allied Health Sciences, School of Health Sciences, the Apollo University, Murukambattu, Chittoor, India; ^5^Saveetha College of Physiotherapy, Saveetha Institute of Medical and Technical Sciences, Chennai, India; ^6^Department of Pharmaceutical Chemistry, Apollo Institute of Pharmaceutical Sciences, The Apollo University, The Apollo Knowledge City Saketa, Chittoor, India; ^7^School of Social Sciences, The Apollo University, Chittoor, India

**Keywords:** Cell cycle, Colorectal neoplasms, Cyclin-dependent kinases, DNA methylation, Epigenesis, Histone modification

## Abstract

**Purpose:**

Epigenetic dysregulation is increasingly recognized as a fundamental driver of cell cycle checkpoint failure in colorectal cancer. Beyond genetic mutations, epigenetic mechanisms provide a dynamic and potentially reversible layer of control over cyclin-dependent kinase activity and tumor progression. This mechanistic critical review examines how epigenetic alterations reshape cell cycle regulation in colorectal cancer and evaluates their translational relevance.

**Methods:**

This review adopts a critical mechanistic approach, integrating and interpreting evidence from experimental, translational and clinical studies addressing epigenetic regulation of cell cycle control in colorectal cancer. Rather than following a systematic review framework, the literature was evaluated conceptually to identify dominant mechanisms, areas of convergence and unresolved controversies.

**Results:**

Evidence indicates that aberrant DNA methylation, histone modifications and chromatin remodeling converge to repress key cyclin-dependent kinase inhibitors including p16INK4a, p21Cip1 and p27Kip1. These epigenetic alterations sustain cyclin–CDK activity thereby promote bypass of G1/S checkpoint control and facilitate uncontrolled proliferation. Importantly, epigenetic heterogeneity across colorectal tumors contributes to variable therapeutic responses and resistance to both conventional and epigenetic-targeted therapies.

**Conclusion:**

Epigenetic control of cell cycle checkpoints represents a central and therapeutically exploitable mechanism in colorectal cancer. A mechanistic understanding of these regulatory networks highlights opportunities for rational combination strategies and precision-based interventions while underscoring current knowledge gaps that warrant further investigation.

## Introduction

###  Global Burden of Colorectal Cancer

 Colorectal cancer (CRC) constitutes a significant global health burden and continues to rank among the leading malignancies in both incidence and mortality, with recent GLOBOCAN estimates reporting nearly 1.9 million new cases and over 900,000 deaths annually.^1,2^ Although historically more prevalent in high-income regions, the incidence of CRC is increasingly rising in low- and middle-income countries, largely driven by transitions in lifestyle patterns including dietary habits, reduced physical activity, obesity and demographic aging.^3–6^ Notwithstanding improvements in screening and therapeutic modalities, CRC remains associated with considerable morbidity and mortality particularly in advanced disease stages.^7,8^ Additionally, the growing incidence of early-onset CRC highlights the contribution of distinct biological and environmental influences in younger populations.^9–11^ At the molecular level, colorectal carcinogenesis is driven by complex interactions between genetic alterations and epigenetic dysregulation that collectively disrupt cellular homeostasis. Aberrant control of key regulatory pathways, particularly those governing cell cycle progression and checkpoint integrity, plays a central role in tumor initiation and progression. Collectively, these trends emphasize the necessity of elucidating the underlying molecular mechanisms of colorectal tumorigenesis, with particular focus on pathways governing cell cycle regulation and epigenetic modifications which may provide critical insights for the development of targeted and mechanism-based therapeutic strategies.^12^

###  Literature Identification and Critical Analytical Approach

 This article is presented as a mechanistic critical review synthesizing published evidence on epigenetic regulation of cell cycle control in colorectal cancer. Relevant experimental, translational and clinical studies were identified through targeted literature exploration and evaluated using a conceptual and analytical framework.

 A structured literature search was conducted using electronic databases including PubMed, Scopus and Web of Science. Key search terms included “colorectal cancer”, “epigenetics”, “DNA methylation”, “histone modification”, “non-coding RNA”, “cell cycle regulation” and “cyclin-dependent kinases”. Studies published in English focusing on molecular mechanisms, translational relevance and therapeutic implications were considered. Priority was given to recent high-impact studies and seminal reports in the field. This review does not follow PRISMA guidelines, does not employ formal risk-of-bias assessment, and is not intended as a systematic review or meta-analysis.

###  Importance of Cell Cycle Dysregulation in Colorectal Cancer

 Sustained and uncontrolled cellular proliferation is a defining hallmark of cancer and dysregulation of the cell cycle plays a central role in colorectal tumor initiation and progression.^13^ Under normal physiological conditions, cell cycle progression is governed by a tightly coordinated network of cyclins, cyclin-dependent kinases (CDKs) and CDK inhibitors that regulate orderly transitions through the G1, S, G2 and M phases, thereby preserving genomic integrity and ensuring controlled cell division.^14,15^ Critical surveillance checkpoints, particularly at the G1/S and G2/M transitions, function to prevent replication and propagation of damaged DNA.^16^

 In colorectal cancer, this finely tuned regulatory system is frequently disrupted. Aberrant overexpression of cyclins such as cyclin D1, constitutive activation of CDKs and functional loss or epigenetic silencing of CDK inhibitors including p16^INK4a^, p21^Cip1^ and p27^Kip1^ collectively drive unchecked cell cycle progression.^17,18^ These alterations promote uncontrolled proliferation, chromosomal instability and progressive accumulation of oncogenic mutations, thereby accelerating tumor growth and disease progression.^19^ (Figure 1)

**Figure 1:**
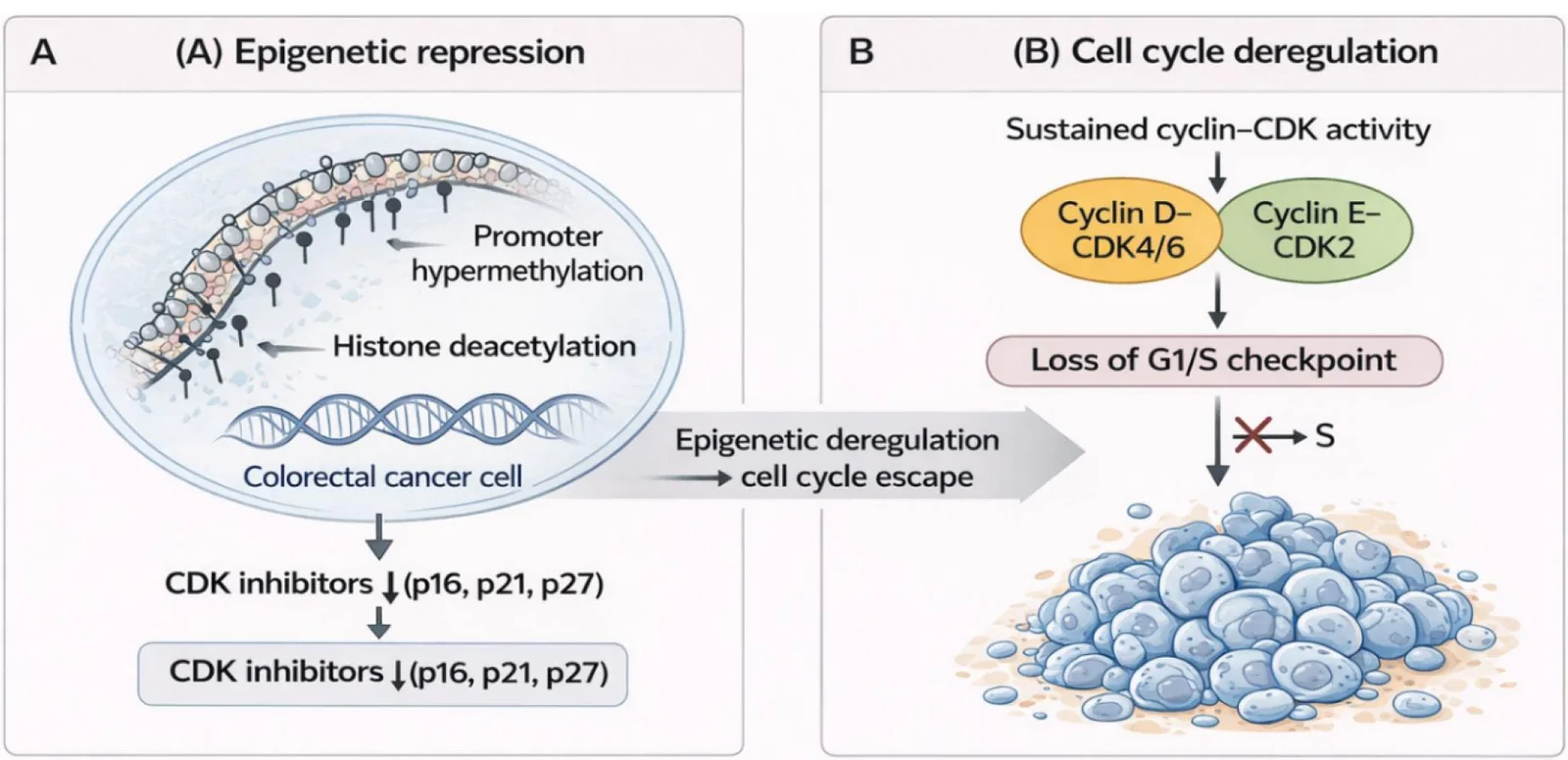
Epigenetic regulation of cell cycle checkpoints in colorectal cancer. Epigenetic alterations, including promoter hypermethylation and aberrant histone modifications, suppress transcription of cyclin-dependent kinase inhibitors such as p16^INK4a^, p21^Cip1^ and p27^Kip1^. This repression results in sustained activation of cyclin–CDK complexes, bypass of the G1/S checkpoint and uncontrolled cellular proliferation characteristic of colorectal tumorigenesis

 Genomic instability arising from defective checkpoint control further fuels tumor evolution and intratumoral heterogeneity. Moreover, impaired cell cycle regulation contributes to resistance against cytotoxic chemotherapy and radiotherapy, as defective checkpoints allow cancer cells to evade apoptosis and survive genotoxic stress.^20,21^ Consequently, delineating the upstream regulatory mechanisms governing cell cycle dysregulation has become essential for identifying novel therapeutic targets in colorectal cancer.^22^ (Table 1)

**Table 1:** Summary of key epigenetic alterations influencing cell cycle checkpoint disruption during colorectal cancer progression

**CRC stage / context**	**Key epigenetic alteration**	**Primary target(s)**	**Checkpoint / pathway impact**	**Functional outcome**
Early adenoma formation	Promoter hypermethylation	CDKN2A (p16^INK4a^)	G1/S checkpoint bypass	Enhanced proliferation and clonal expansion (e.g.,CDKN2A/p16INK4a silencing, SFRP1-mediated Wnt activation)
Progressive dysplasia	Histone deacetylation	CDKN1A (p21^Cip1^)	Reduced CDK inhibition	Increased cell cycle progression and checkpoint evasion *(e.g., CDKN1A/p21Cip1 repression, GADD45A downregulation)*
Advanced tumor growth	Chromatin remodeling dysregulation	CDKN1B (p27^Kip1^)	Cyclin–CDK activation	Loss of growth control and sustained proliferation *(e.g., CDKN1B/p27Kip1 downregulation, CCNE1 overexpression)*
Metastatic progression	Global hypomethylation	Genome-wide instability loci	Chromosomal instability	Increased invasiveness and phenotypic plasticity *(e.g., CDH1 silencing promoting EMT, CXCR4 activation)*
Therapy-resistant subclones	Epigenetic plasticity	Multiple tumor suppressor pathways	Checkpoint adaptation	Resistance to chemo/radiotherapy and survival advantage *(e.g., ABCB1 upregulation, BCL2L1-mediated apoptosis resistance)*

###  Emergence of Epigenetics as a Regulatory Layer

 Beyond irreversible genetic mutations, epigenetic alterations have emerged as a pivotal regulatory layer that shapes gene expression and cellular behavior in colorectal cancer.^23^ Epigenetics refers to heritable yet reversible modifications that influence chromatin architecture and gene expression without altering the DNA sequence, predominantly mediated through DNA methylation, histone modifications, and regulatory non-coding RNAs.^24-26^ These mechanisms are essential for normal development and tissue homeostasis and their disruption is increasingly regarded as a fundamental contributor to malignant transformation.^27^

 Colorectal cancer exhibits extensive epigenetic reprogramming, characterized by global DNA hypomethylation, promoter-specific hypermethylation of tumor suppressor genes, abnormal histone acetylation and methylation signatures and dysregulated microRNA expression.^28-30^ Such epigenetic remodeling not only alters transcriptional output but also provides colorectal tumor cells with adaptive plasticity that supports proliferation, survival, and therapeutic resistance. Large-scale epigenomic and transcriptomic profiling studies, including those conducted by The Cancer Genome Atlas, have identified distinct molecular and epigenetic subtypes of colorectal cancer with characteristic DNA methylation and chromatin modification signatures that correlate with tumor behavior, disease progression and therapeutic response.^30,31^

 Epigenetic mechanisms directly modulate the transcription of key cell cycle regulators, thereby linking environmental exposures and oncogenic signaling pathways to cell cycle control.^32-34^ Importantly, epigenetic alterations often occur early during colorectal tumorigenesis and may precede irreversible genetic mutations, suggesting a critical role in disease initiation.^35,36^ In contrast to fixed genetic lesions, epigenetic dysregulation is inherently dynamic and may be pharmacologically reprogrammed, positioning it as a promising target for therapeutic intervention.. Increasing evidence indicates that pharmacological modulation of epigenetic regulators, such as DNA methyltransferases and histone deacetylases, can restore cell cycle checkpoint function and enhance sensitivity to conventional anticancer therapies.^37-39^ Collectively, these observations highlight epigenetic regulation of cell cycle control as a promising and rational therapeutic avenue in colorectal cancer management.^40-49^

 The overall conceptual framework and organization of this review are aligned with contemporary review articles in the field, emphasizing mechanistic insight and translational relevance in biomedical research.^50^

###  Cell Cycle Regulation in Colorectal Cancer

####  a) Overview of the Cell Cycle Machinery

 The eukaryotic cell cycle represents a highly ordered regulatory program designed to coordinate genome duplication with accurate chromosomal partitioning during cell division. Progression through this program is conventionally organized into four phases G1, S, G2 and M and is driven by cyclical fluctuations in cyclin-dependent kinase (CDK) activity.^51,52^ CDKs exert their function through association with phase-specific cyclins, generating enzymatic complexes whose activation is precisely timed to enforce directional movement through the cell cycle.^53^

 Distinct cyclin–CDK assemblies orchestrate key transition points. Cyclin D–CDK4/6 complexes initiate early G1 progression, whereas cyclin E–CDK2 serves as a critical regulator of the G1/S checkpoint. DNA replication during S phase is primarily supported by cyclin A–CDK2, while cyclin B–CDK1 activity triggers mitotic entry.^54,55^ Because CDKs act as central gatekeepers of replication and division, stringent regulation of their activation is essential. Dysregulated CDK signaling can induce replication stress, promote DNA damage accumulation and accelerate chromosomal instability.^56^

 Multiple regulatory mechanisms restrain CDK activity, including inhibitory phosphorylation, ubiquitin-mediated degradation of cyclins, and inhibition by cyclin-dependent kinase inhibitors (CDKIs).^57^ These safeguards collectively ensure that cell cycle progression occurs only under conditions favorable for genomic integrity.

####  b) Cell Cycle Checkpoints and Their Disruption in Colorectal Cancer

 Cell cycle checkpoints act as regulatory surveillance barriers that assess genomic integrity and cellular preparedness before allowing progression through the cell cycle. The G1/S checkpoint restricts entry into S phase to prevent duplication of damaged DNA, whereas the G2/M checkpoint verifies that DNA replication is complete and that repair processes have been adequately executed before mitotic entry.^58^ These checkpoints are governed by tumor suppressor pathways, most notably the p53–p21 axis and the retinoblastoma (RB) pathway.^59^

 In colorectal cancer, checkpoint integrity is frequently compromised through genetic and epigenetic alterations. Functional inactivation of p53, deregulation of RB signaling, and aberrant activation of CDKs collectively enable cancer cells to bypass checkpoint control.^60,61^ This permissive environment allows proliferation despite genomic damage, accelerating mutation accumulation and tumor evolution.^62^ Sustained checkpoint failure also contributes to chromosomal instability, a defining molecular feature of colorectal cancer associated with aggressive disease and poor prognosis.^63^

 Checkpoint dysfunction has important therapeutic implications. Colorectal cancer cells with defective checkpoints often rely on residual DNA damage response pathways for survival, rendering them selectively vulnerable to therapies targeting replication stress and mitotic control.^64,65^

###  Epigenetic Mechanisms in Colorectal Cancer

####  a) DNA Methylation and Transcriptional Control

 DNA methylation refers to the enzymatic transfer of a methyl group onto cytosine bases, predominantly within CpG dinucleotides, a reaction mediated by DNA methyltransferases. This modification is essential for physiological gene regulation, maintaining chromosomal integrity, and ensuring stable epigenomic patterning during development.^66^ In colorectal cancer, methylation landscapes are frequently distorted, displaying widespread genomic hypomethylation alongside focal CpG island hypermethylation at promoter regions of tumor suppressor genes.^67,68^ Functionally, promoter-directed hypermethylation creates a transcriptionally repressive environment that restricts expression of genes governing checkpoint enforcement, programmed cell death, and DNA damage repair. In colorectal carcinogenesis, such silencing events often arise early and may act as initiating molecular switches that predispose cells to unchecked proliferation and progressive malignant transformation.^69^ A well-recognized manifestation of this phenomenon is the CpG island methylator phenotype, which defines a distinct CRC subtype marked by extensive promoter hypermethylation and characteristic clinicopathological associations.^70,71^

####  b) Histone Modifications and Chromatin Remodeling

 Histones serve as dynamic regulatory scaffolds, and their post-translational modification including acetylation, methylation, phosphorylation and ubiquitination shapes chromatin architecture and transcriptional permissiveness.^72^ Among these modifications, histone acetylation typically promotes chromatin relaxation and facilitates transcription, whereas histone deacetylation promotes a compact chromatin state and transcriptional repression. These processes are regulated through the opposing actions of histone acetyltransferases and histone deacetylases. In colorectal cancer, aberrant deacetylase activity has been linked to inappropriate chromatin compaction and repression of tumor suppressor pathways, including those required for cell cycle arrest.^73,74^ Histone methylation introduces an additional regulatory dimension, as its transcriptional impact depends on both the specific residue modified and the extent of methyl group deposition. Dysregulated expression or catalytic activity of histone methyltransferases and demethylases has been implicated in colorectal tumorigenesis through epigenomic reprogramming of genes controlling proliferation, checkpoint signaling, and cellular fate decisions.^75,76^ (Figure 2)

**Figure 2:**
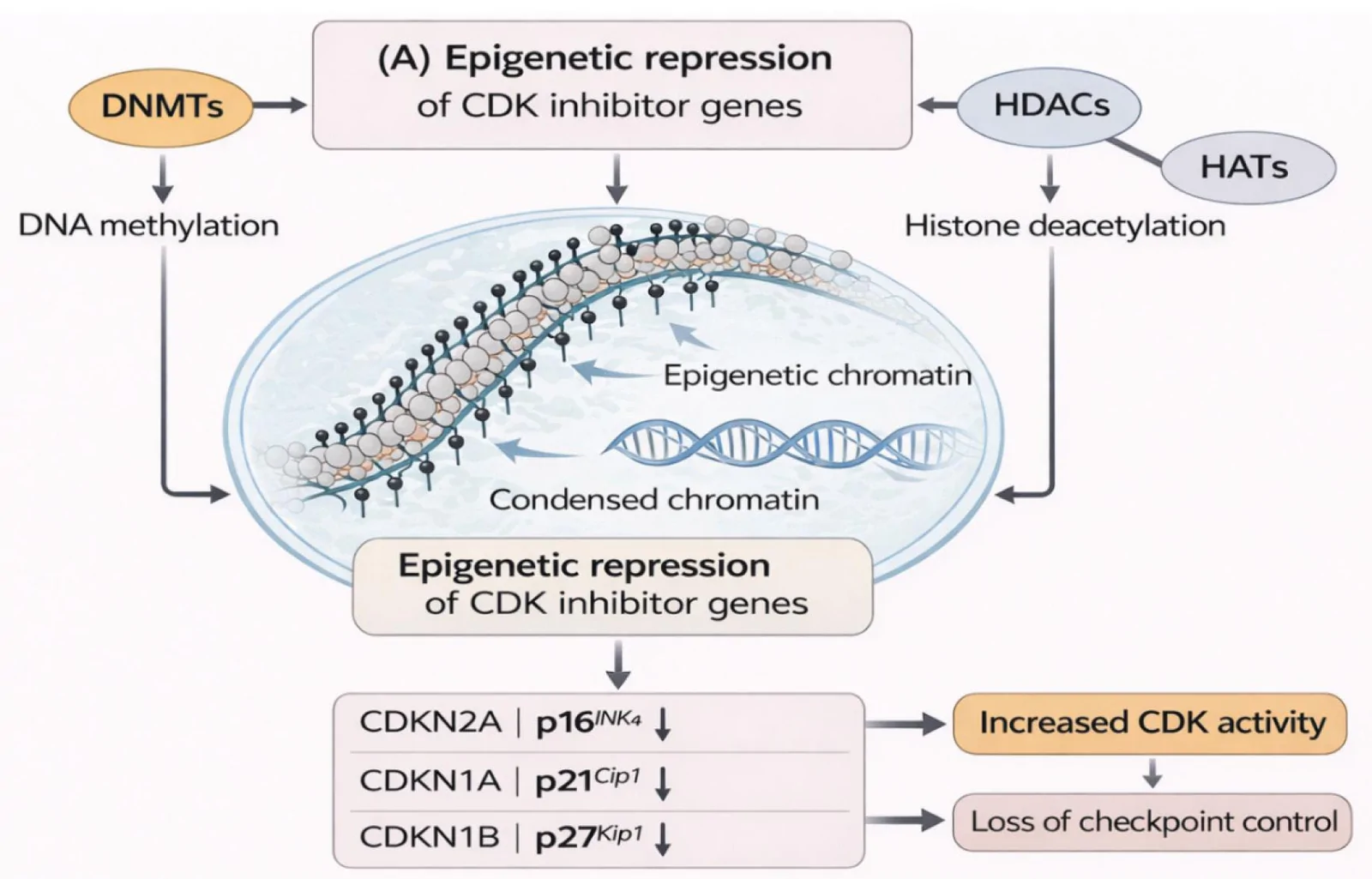
Molecular epigenetic mechanisms governing cyclin-dependent kinase inhibitor expression in colorectal cancer. DNA methylation and histone deacetylation cooperate to induce chromatin condensation and transcriptional repression of CDK inhibitor genes, whereas epigenetic reprogramming can restore chromatin accessibility and checkpoint integrity

####  c) Role of Non-Coding RNAs

 Non-coding RNAs constitute a critical epigenetic regulatory axis capable of modulating gene expression at both transcriptional and post-transcriptional levels (Table 2). MicroRNAs can suppress cell cycle progression by directly targeting mRNAs encoding cyclins, cyclin-dependent kinases and checkpoint inhibitory proteins, thereby reshaping proliferative signaling in colorectal cancer cells.^77,78^ In parallel, long non-coding RNAs contribute to epigenetic remodeling by functioning as molecular scaffolds or guides that recruit chromatin-modifying complexes to defined genomic loci, enabling locus-specific transcriptional activation or repression.^79,80^

**Table 2:** Representative non-coding RNAs modulating epigenetic regulators and cell cycle signaling in colorectal cancer

**Non-coding RNA type**	**Representative molecule**	**Epigenetic mechanism**	**Cell cycle target**	**Functional implication in CRC**
microRNA	miR-34a	p53-regulated miRNA that suppresses CDK6 and cyclin D1, enforcing cell cycle arrest	CDK6, cyclin D1	G1 arrest and tumor suppression
microRNA	miR-21	Targets tumor suppressors such as PTEN and PDCD4, leading to enhanced proliferation and reduced apoptosis	PTEN, p21 axis	Enhanced proliferation and survival
lncRNA	HOTAIR	Recruits PRC2 complex to mediate H3K27 trimethylation and gene silencing	p21/p27-associated pathways	Promotion of invasion and proliferation
lncRNA	MALAT1	Regulates alternative splicing and transcriptional control of cell cycle genes	Cell cycle regulators	Enhanced proliferation and metastatic potential
circRNA	circHIPK3	Acts as a ceRNA by sponging miR-124 and miR-7, promoting proliferation-associated gene expression.	CDK inhibitors	Cell cycle progression and growth advantage

 Despite substantial evidence implicating DNA methylation, histone modifications and chromatin remodeling in colorectal cancer progression, the relative contribution of individual epigenetic mechanisms remains context dependent. Studies differ in the extent to which specific epigenetic alterations function as primary drivers versus secondary adaptations to oncogenic signaling, and in some cases, opposing epigenetic states have been reported across molecular subtypes. This variability likely reflects tumor heterogeneity, differences in experimental models, and dynamic temporal regulation during disease evolution. Consequently, epigenetic mechanisms should not be viewed as uniform oncogenic switches but rather as flexible regulatory processes whose functional impact depends on cellular context and disease stage.

###  Epigenetic Regulation of Cell Cycle Control

 Epigenetic regulation is pivotal in orchestrating cell cycle progression by controlling chromatin structure and transcriptional access to key regulators such as cyclins, cyclin-dependent kinases (CDKs), and their endogenous inhibitors. In colorectal cancer, these epigenetic modifications act as dynamic molecular switches that integrate environmental influences, oncogenic pathways, and cellular stress signals, ultimately reprogramming cell cycle checkpoints.^81,82^ Unlike genetic mutations, epigenetic alterations occur without changing the underlying DNA sequence. but exert profound effects on transcriptional output, thereby enabling cancer cells to sustain proliferative signaling while retaining phenotypic plasticity.^83^

 Aberrant DNA methylation, histone modification patterns and non-coding RNA networks converge to suppress cell cycle checkpoint regulators and enhance cyclin–CDK activity. This coordinated epigenetic reprogramming permits colorectal cancer cells to bypass growth-inhibitory signals, evade senescence and tolerate genomic instability.^84,85^ Importantly, epigenetic control of cell cycle is context dependent and reversible, positioning it as a critical interface between tumor biology and therapeutic intervention.

###  Epigenetic Control of CDK Inhibitors

 Cyclin-dependent kinase inhibitors (CDKIs) serve as key molecular brakes that restrain CDK activity and preserve checkpoint fidelity. Among these, p16^INK4a^, p21^Cip1^ and p27^Kip1^ are central regulators of G1/S phase transition and are frequent targets of epigenetic silencing in colorectal cancer.^86^

 p16^INK4a^ selectively inhibits CDK4 and CDK6, preventing phosphorylation of the retinoblastoma protein and enforcing G1-phase arrest. In colorectal cancer, promoter hypermethylation of the *CDKN2A* locus is a common epigenetic event that leads to transcriptional silencing of p16^INK4a^.^87^ Loss of p16^INK4a^ expression results in sustained cyclin D–CDK4/6 activity, enabling unchecked cell cycle entry despite growth-inhibitory cues.

 p21^Cip1^, a downstream effector of p53 signaling, functions as a broad-spectrum CDK inhibitor linking DNA damage responses to cell cycle arrest. Epigenetic repression of *CDKN1A* through histone deacetylation or altered chromatin occupancy disrupts p21^Cip1^ mediated checkpoint control, thereby decoupling DNA damage sensing from proliferative restraint.^88^ This dysregulation contributes to resistance against genotoxic therapies and facilitates survival under replicative stress.

 p27^Kip1^ primarily inhibits cyclin E–CDK2 and cyclin A–CDK2 complexes, regulating late G1 and S-phase progression. Reduced expression of p27^Kip1^ in colorectal cancer is frequently driven by epigenetic mechanisms, including microRNA mediated repression and chromatin remodeling, rather than direct genetic alterations.^89^ Loss of p27^Kip1^ promotes sustained CDK2 activity and correlates with aggressive tumor behavior and poor clinical outcomes.

 Collectively, epigenetic suppression of CDK inhibitors dismantles checkpoint control and establishes a permissive proliferative state, highlighting these molecules as critical nodes linking chromatin dynamics to cell cycle deregulation in colorectal cancer.^90^

 While epigenetic silencing of cyclin-dependent kinase inhibitors represents a consistent mechanistic feature of colorectal cancer, translation of these insights into clinical practice remains limited. Reduced expression of p16^INK4a^, p21^Cip1^ and p27^Kip1^ has been variably associated with aggressive tumor behavior, therapeutic resistance and adverse outcomes; however, these relationships are not uniformly predictive across patient cohorts. This inconsistency underscores the influence of epigenetic context and co-existing molecular alterations. Integrating CDK inhibitor status with broader epigenetic and transcriptomic profiles may therefore be necessary to realize their full prognostic and therapeutic potential.

###  Therapeutic Implications of Targeting Epigenetic Regulators

 The reversible nature of epigenetic modifications provides a compelling biological rationale for therapeutic intervention in colorectal cancer. Unlike permanent genetic alterations, epigenetic changes can be pharmacologically modulated, enabling restoration of transcriptional programs governing cell cycle arrest, apoptosis, and cellular differentiation.^91,92^ Consequently, epigenetic therapies offer the potential to re-establish tumor suppressor function rather than merely suppress downstream oncogenic signaling pathways. DNA methyltransferase inhibitors represent one of the most extensively investigated classes of epigenetic agents. Aberrant promoter hypermethylation is a frequent mechanism underlying transcriptional silencing of cyclin-dependent kinase inhibitors in colorectal cancer.^93^ Pharmacological inhibition of DNMTs can reverse this silencing, leading to re-expression of genes such as CDKN2A and restoration of G1/S checkpoint control.^94^ Preclinical studies consistently demonstrate that demethylating agents suppress proliferation, induce cell cycle arrest, and enhance apoptotic sensitivity in colorectal cancer models.^95^

 Histone deacetylase inhibitors constitute another major class of epigenetic therapeutics with direct relevance to cell cycle regulation. By promoting histone acetylation and chromatin relaxation, HDAC inhibitors facilitate transcriptional activation of growth-inhibitory genes, including p21^Cip1^ and p27^Kip1^.^96^ This reactivation attenuates CDK activity and reinforces cell cycle checkpoint control. Beyond cytostatic effects, HDAC inhibitors modulate multiple oncogenic pathways, including DNA damage response signaling and apoptotic machinery.^97^ Importantly, accumulating evidence suggests that epigenetic therapies may exert their greatest clinical benefit when employed as part of combination strategies rather than as monotherapies (Figure 3).

**Figure 3:**
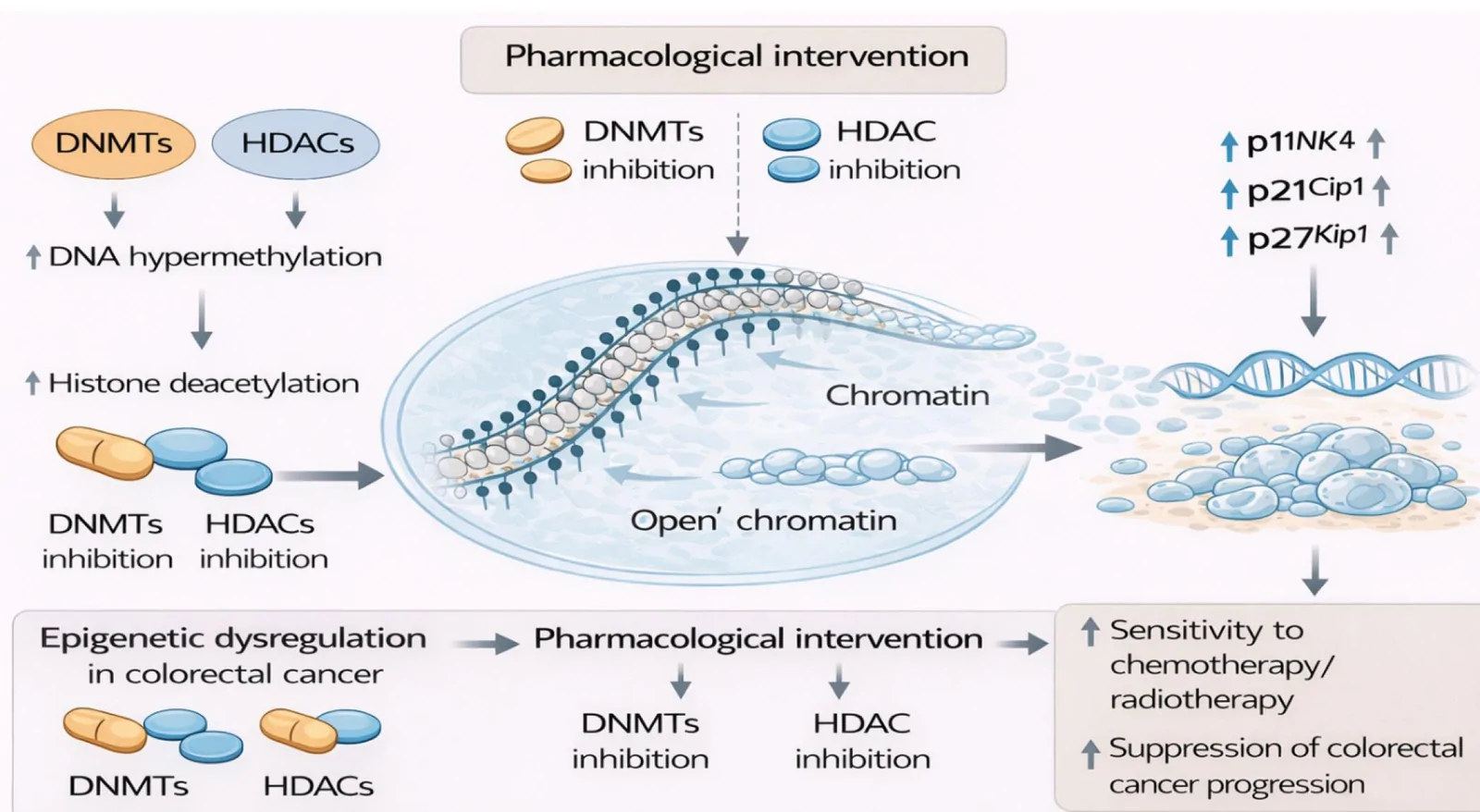
Therapeutic targeting of the epigenetic–cell cycle axis in colorectal cancer. Pharmacological inhibition of DNA methyltransferases and histone deacetylases restores tumor suppressor gene expression, reinforces cell cycle checkpoints, and enhances sensitivity to conventional anticancer therapies

 Epigenetic priming has been shown to sensitize colorectal cancer cells to chemotherapy, radiotherapy, and targeted agents by restoring apoptotic competence and checkpoint integrity.^98^ Combination regimens integrating epigenetic modulators with CDK4/6 inhibitors or immune checkpoint inhibitors are therefore emerging as promising approaches to overcome therapeutic resistance.^99,100^

 Despite encouraging preclinical findings, clinical translation of epigenetic therapies in colorectal cancer remains challenging. Limited target specificity, dose-limiting toxicities, and marked interpatient heterogeneity have constrained their widespread adoption.^101^ These limitations underscore the need for robust predictive biomarkers to guide patient selection and optimize rational therapeutic combinations

###  Challenges, Knowledge Gaps, and Future Directions

 Although significant advances have been achieved in understanding how epigenetic mechanisms regulate cell cycle progression, several key challenges still persist (Table 3). One major limitation is the context-dependent nature of epigenetic alterations. The functional consequences of DNA methylation or histone modification are influenced by tumor subtype, cellular differentiation state and micro-environmental cues, complicating therapeutic targeting.^102^

**Table 3:** Key barriers limiting clinical translation of epigenetic therapies and strategies to improve colorectal cancer treatment outcomes

**Challenge domain**	**Underlying epigenetic factor**	**Clinical consequence**	**Therapeutic implication**	**Future direction**
Interpatient heterogeneity	Subtype-specific methylation signatures	Variable response to epigenetic drugs	Need biomarker-guided selection	Integrate methylome profiling into stratification
Intratumoral heterogeneity	Subclonal chromatin state diversity	Emergence of resistant subpopulations	Combination therapy requirement	Single-cell epigenomics-guided targeting
Off-target toxicity	Global chromatin modulation	Dose-limiting adverse effects	Restricted therapeutic window	Develop selective epigenetic inhibitors
Epigenetic plasticity	Dynamic reversible switching	Transient drug sensitivity	Adaptive resistance	Sequential/priming-based regimens
Lack of predictive biomarkers	Complex multi-layer regulation	Uncertain patient benefit	Trial failures	Multi-omic biomarker panels

 Another unresolved issue is the lack of specificity of current epigenetic drugs. Global modulation of DNA methylation or histone acetylation may inadvertently affect normal cells, leading to off-target effects and toxicity.^103^ The development of next-generation epigenetic therapies with locus-specific or enzyme-selective activity is therefore a critical unmet need.^104^

 Tumor heterogeneity further complicates therapeutic intervention. Epigenetic landscapes vary not only between patients but also within individual tumors, enabling adaptive resistance and disease progression.^105^ Single cell epigenomic technologies have begun to reveal this heterogeneity, but integration of these insights into clinical decision-making remains in its infancy.^106^

 Future research should focus on defining epigenetic signatures that predict response to therapy, identifying combinatorial vulnerabilities linking epigenetic dysregulation to cell cycle dependence, and developing precision epigenetic editing approaches.^107^ Recent developments in CRISPR-based epigenome editing and integrated multi-omics approaches provide powerful new avenues to unravel causal links between chromatin dynamics and cell cycle regulation (Figure 4).^108^

**Figure 4:**
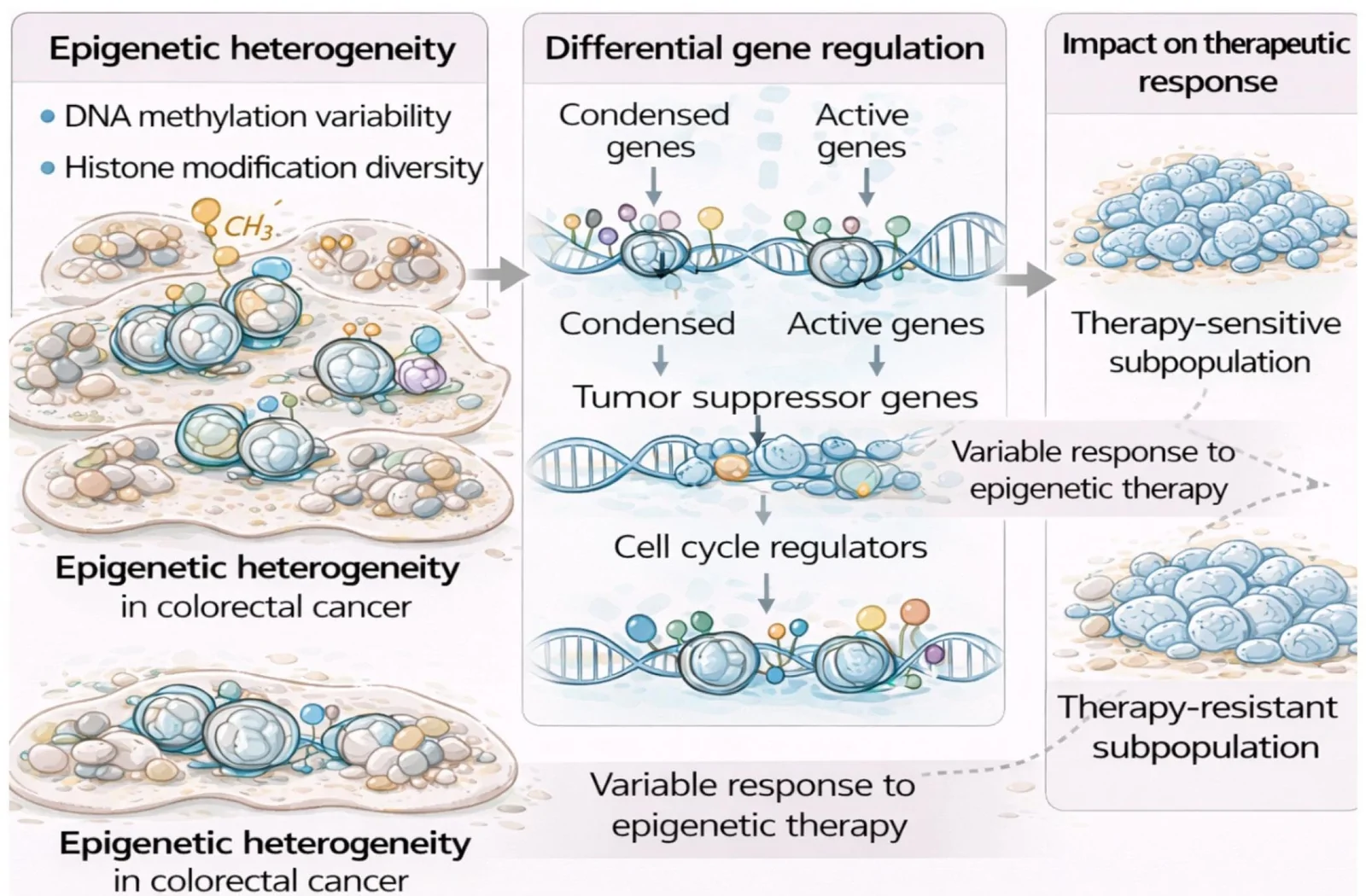
Integrative model illustrating the interplay between epigenetic dysregulation, cell cycle control, and therapeutic response in colorectal cancer. Epigenetic alterations function as upstream regulators of proliferative competence and therapeutic sensitivity

 Ultimately, translating epigenetic insights into durable clinical benefit will require a shift toward mechanism-driven, biomarker-guided therapeutic strategies that account for tumor heterogeneity and dynamic epigenetic plasticity.^109^ Such approaches hold promise for improving outcomes in colorectal cancer by exploiting the reversible nature of epigenetic regulation.^110^

## Conclusion

 Colorectal cancer illustrates how malignant progression can arise not only from fixed genetic lesions but also from dynamic and reversible disruptions in regulatory architecture. The evidence synthesized in this review positions epigenetic control of the cell cycle as a central organizing principle in colorectal tumor biology, rather than a secondary or accessory process. Through coordinated modulation of chromatin accessibility and transcriptional output, epigenetic alterations recalibrate proliferative thresholds, enabling colorectal cancer cells to bypass checkpoint constraints while maintaining adaptability to environmental and therapeutic pressures.

 A defining feature of this regulatory rewiring is the epigenetic attenuation of cyclin-dependent kinase inhibitors such as p16^INK4a^, p21^Cip1^ and p27^Kip1^. Loss of these molecular brakes does not simply accelerate cell division; it fundamentally alters how colorectal cancer cells interpret DNA damage, metabolic stress, and growth-inhibitory cues. In this context, the cell cycle shifts from a regulated decision-making framework to a permissive, self-reinforcing driver of tumor evolution. Importantly, such changes often precede irreversible genetic instability, suggesting that epigenetic dysregulation functions as an early architect of malignant behavior.

 From a therapeutic standpoint, epigenetic regulation of the cell cycle represents an opportunity to intervene upstream of established oncogenic dependency. Rather than targeting isolated signaling nodes, epigenetic strategies offer the potential to restore regulatory balance across entire checkpoint networks. Realizing this potential, however, will require moving beyond global chromatin modulation toward precision approaches that account for tumor subtype, epigenetic context, and intratumoral heterogeneity.

 Looking forward, the convergence of epigenomics, cell cycle biology, and systems-level analytics provides a pathway toward redefining therapeutic vulnerability in colorectal cancer. By conceptualizing the epigenome as an adaptive regulator of proliferative competence rather than a static layer of modification, future research may shift clinical paradigms from suppressing tumor growth to reinstating lost regulatory control, enabling more durable and context-aware strategies for colorectal cancer management.

## Competing Interests

 The authors confirm that there are no financial or non-financial conflicts of interest related to this manuscript.

## Ethical Approval

 This manuscript is a literature-based review, includes no human / animal research and no identifiable personal data; therefore, ethical approval was not applicable.
